# Prevalence and Associated Factors of Sarcopenic Obesity in the Community Elderly: Meta-Analysis and Systematic Review

**DOI:** 10.3390/nu18081267

**Published:** 2026-04-16

**Authors:** Xinyue Zhang, Ying Fan, Lijiangshan Hua, Yitao Zhou, Qiuhua Sun

**Affiliations:** School of Nursing, Zhejiang Chinese Medical University, Hangzhou 310053, China; 13819395798@163.com (X.Z.); 15172570436@163.com (Y.F.); hljs366@zcmu.edu.cn (L.H.); zhouyitao1124@163.com (Y.Z.)

**Keywords:** community, sarcopenic obesity, prevalence, associated factors, prognosis, meta-analysis

## Abstract

***Background***: Through a meta-analysis and systematic review, the present study aimed to evaluate the prevalence, associated factors and prognosis of sarcopenic obesity in the elderly in the community. ***Methods***: From database inception to 31 December 2025, this study performed a full database of PubMed, Web of Science, Embase, the Cochrane Library, CINAHL, CNKI, CBM, WANFANG, and VIP database. Two researchers undertook a systematic process of data extraction and literature quality evaluation. Stata 17 was used to evaluate the prevalence, associated factors and prognosis of sarcopenic obesity in the elderly in the community. ***Results***: Our study included a total of 37 sources, encompassing 80,337 elderly individuals in the community. The results showed that the sarcopenic obesity prevalence in this population was 10%(95%*CI*: 8–11%), with a 95% prediction interval of 1.4–31.2%, and its occurrence was related to multiple associated factors such as age (OR = 1.83, 95%*CI*: 1.21–2.76), male (OR = 3.38, 95%*CI*: 1.53–7.49), low physical activity (OR = 1.56, 95%*CI*: 1.13–2.16), moderate-to-high physical activity (OR = 0.62, 95%*CI*: 0.51–0.77), low income (OR = 1.71, 95%*CI*: 1.04–2.83), unemployment (OR = 1.88, 95%*CI*: 1.29–2.75) and insufficient energy intake (OR = 1.23, 95%*CI*: 1.02–1.50). The poor prognosis of sarcopenic obesity in the elderly in the community, including falls, disability, increased risk of hospitalization, and death, seriously affects their quality of life. ***Conclusions***: The prevalence of sarcopenic obesity in the elderly in the community is relatively high. Age, gender, income level and other factors are closely associated with the occurrence of sarcopenic obesity and can lead to serious adverse consequences. It is recommended that primary medical institutions should focus on people at a high risk of sarcopenic obesity. Community medical personnel can formulate targeted prevention and control measures according to their associated factors to achieve early screening, diagnosis and intervention.

## 1. Introduction

Sarcopenic obesity (SO) is a condition in which sarcopenia and obesity coexist, constituting a significant global public health concern. As early as 1996, Heber et al. began a preliminary exploration of this problem [1]. In 2000, Baumgartner clarified its clinical definition [2]. Currently, SO is widely regarded as an independent clinical entity. Its main characteristics include a reduction in skeletal muscle mass and function, coexisting with an excessive accumulation of fat [3]. Existing studies show that SO is an important prognostic factor for disability and mortality, and can also significantly increase the risk of obesity-associated comorbidities [4,5]. With the population aging and rising obesity rates, the SO prevalence among the elderly is increasing steadily. The trend of this disease has serious health consequences, not only increasing the risk of physical dysfunction, metabolic disorders, and cardiovascular diseases but also significantly increasing the incidence of other adverse outcomes such as falls, disability, and death. Ultimately, it poses challenges to both individual quality of life and the public health system.

In recent years, SO has increasingly become a focus of academic research. Existing studies primarily concentrate on the analysis of prevalence and associated factors, and most of them are small-sample cross-sectional studies, which limits the generalizability of their conclusions. For example, a cross-sectional study conducted by Gee et al. in South Korea found that the prevalence of SO in the elderly in the community was 1.08% [6]. In research conducted by Moonkyoung et al., a prevalence rate of 3.54% was identified [7]. Conversely, another study revealed that the prevalence reached 29.3% among 270 elderly individuals in a Brazilian community [8]. Therefore, there are significant differences in the reported SO prevalence among the elderly across different studies and communities. It is necessary to meta-analyze the overall disease prevalence for a more reliable estimate for this population.

Currently, some relevant scholars have discussed the associated factors and prognosis of SO among the elderly in the community. However, there is a considerable heterogeneity across the existing studies, and the conclusions are still inconsistent. To illustrate, although Jina et al. found that insufficient protein intake is not a risk factor for SO [9], many other studies have drawn the opposite conclusion [8,10,11]. Similarly, Moonkyoung et al. identified smoking as a risk factor [8], whereas other findings do not support this association [7]. Although some scholars have conducted a meta-analysis of SO, the comprehensive epidemiological evidence of SO in the elderly in the community is limited, and some studies have certain deficiencies. Guimaraes et al. performed a meta-analysis and systematic evaluation of the relevant factors of SO, but their research was limited to Brazil, which did not pay attention to the elderly and failed to comprehensively evaluate the prevalence and prognosis [12]. Other studies, such as the global prevalence study by Qianqian et al., did not specifically evaluate community-dwelling populations or analyze associated factors and prognostic outcomes [13]. Notably, there is still a lack of systematic reviews specifically addressing the prognosis of SO in community-dwelling older adults. Therefore, this study aims to comprehensively evaluate the prevalence, associated factors, and prognosis of SO in this population through meta-analysis and systematic review, providing scientific and practical decision-making support for community health service institutions and primary healthcare professionals in conducting relevant screening efforts. This will also contribute to the early identification of high-risk groups and offer evidence-based recommendations to improve awareness, control, and treatment of SO.

## 2. Materials and Methods

This study was conducted following the guidelines of the Preferred Reporting Items for Systematic Reviews and Meta-Analyses (PRISMA) [14,15]. The study protocol has been registered in PROSPERO under registration number CRD420251184337.

### 2.1. Search Strategy

This study searched PubMed, Web of Science, Embase, the Cochrane Library, CINAHL, China Knowledge Resource Integrated Database (CNKI), Chinese Biomedical Database (CBM), Wanfang Database, and Weipu Database (VIP), and manually screened the reference lists of included studies. As for the search period, it spanned from the inception of each database to 31 December 2025. A combination of Medical Subject Headings terms and free-text keywords was used, with the following search strategy applied: (sarcopenia OR sarcopenic) AND (obesity OR sarcopenic obesity OR obesity, abdominal OR body weight) AND (prevalence OR incidence OR epidemiology OR frequency OR influence factors OR associated factors OR influencing factor OR affecting factor OR risk factor OR predictor OR relevant factor OR related factor OR causality). Table S1 lists the complete strategy.

### 2.2. Inclusion and Exclusion Criteria

The inclusion criteria were as follows: (1) Participants: community-dwelling adults aged ≥60 years. (2) Reported prevalence, influencing factors, or prognosis of SO. (3) SO defined according to international consensus guidelines, such as those from the European Working Group on Sarcopenia in Older People (EWGSOP), Asian Working Group for Sarcopenia (AWGS), European Society for Clinical Nutrition and Metabolism (ESPEN), and European Association for the Study of Obesity (EASO) [16,17,18]. (4) Study design: observational studies.

The exclusion criteria included the following: (1) Duplicate publications or studies with incomplete data. (2) Non-research articles such as systematic reviews, case reports, protocols, animal studies, and conference abstracts. (3) Studies not published in English or Chinese. (4) Sample size < 100. (5) Studies focusing exclusively on specific populations, such as diabetic patients, postmenopausal women, or cohorts consisting solely of males or females.

### 2.3. Data Extraction

The retrieved records were imported into EndNote 21 for deduplication. Two investigators independently conducted the initial screening by evaluating titles and abstracts against the pre-established eligibility criteria. Articles meeting the preliminary criteria underwent full-text review for final assessment. The two researchers then cross-checked their selections. If there were any disagreements, they were addressed through discussion or arbitration by other a third reviewer. Data extraction covered the first author, publication year, country, study design, sample size, muscle measurement tool, age, definition of SO, prevalence, associated factors, and prognosis.

### 2.4. Study Quality Appraisal

The quality of cross-sectional studies was evaluated using the 11-item assessment tool developed by the Agency for Healthcare Research and Quality (AHRQ). Each item is rated as “yes,” “no,” or “unclear,” with scores ranging from 0 to 1 and a maximum total of 11. Studies with a total score ≤ 3 were classified as low-quality [19]. For cohort or case–control studies, the Newcastle-Ottawa Scale (NOS) was employed. The NOS includes eight items that are organized into three domains: selection, comparability, and outcome, with a maximum score of 9. Studies scoring between 0 and 3 were considered low-quality [20]. Any disagreements in quality assessment were resolved through consensus between reviewers or, when necessary, by consultation with other researchers.

### 2.5. Data Analysis

Statistical analyses were performed in Stata 17 (MP 17.0). Pooled prevalence estimates and odds ratios (ORs) with 95% confidence intervals (*CI*s) were calculated. Heterogeneity among included studies was quantified using the *I*^2^ statistic. When *p* ≥ 0.10 or *I*^2^ ≤ 50%, this indicated low or no heterogeneity between the included studies, and a fixed-effects model was applied. In cases where *p* ≤ 0.10 or *I*^2^ ≥ 50%, suggesting significant heterogeneity, a random-effects model was employed. Subgroup analysis and meta-regression were performed to investigate potential sources of heterogeneity. Sensitivity analysis was carried out by using the leave-one-out method and combining model transformation to examine the stability of the pooled results. Publication bias was assessed through Egger’s test and funnel plots. The difference was statistically significant with *p* < 0.05. Because there was little literature on the prognosis of SO in community-dwelling older adults, only descriptive analyses were conducted.

## 3. Results

### 3.1. Study Process

**Figure 1:**
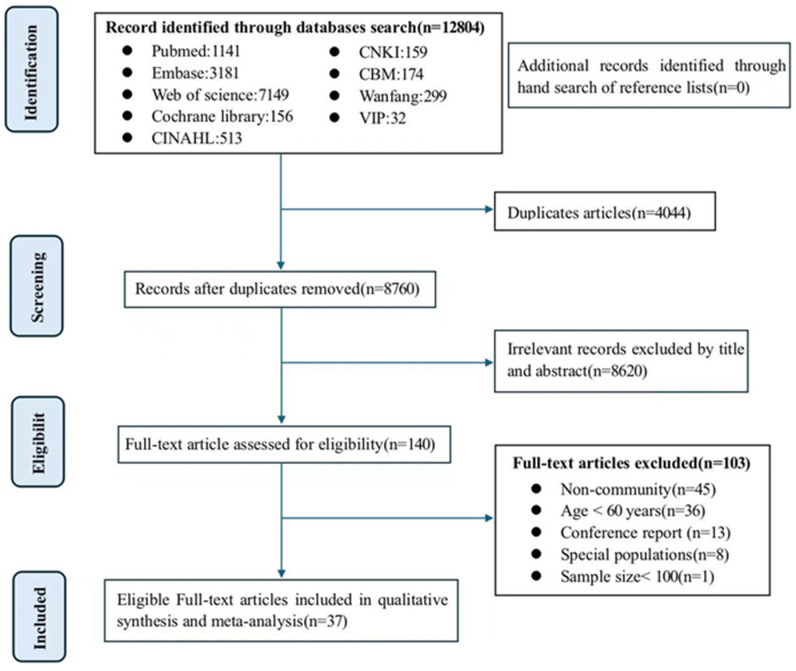
PRISMA flow diagram of the study selection process.

A total of 12,804 records were identified through the initial search, of which 4044 were duplicates, and 140 articles were screened according to eligibility criteria. Upon full-text review, 81 articles were excluded for not meeting age or community-dwelling criteria, 8 were excluded due to ineligible study populations, 13 were excluded as conference reports, and 1 was excluded for having a sample size of less than 100. Ultimately, 37 articles (all published in English) qualified and were incorporated into our study. Figure 1 illustrates the selection process.

### 3.2. Study Characteristics and Quality Assessment

The 37 articles included 80,337 community-dwelling older adults. Regarding study design, two were cohort studies [21,22] and the remaining were cross-sectional studies [6,7,8,9,10,11,23,24,25,26,27,28,29,30,31,32,33,34,35,36,37,38,39,40,41,42,43,44,45,46,47,48,49,50,51]. All studies were of moderate to high quality, with 12 rated as high-quality [6,8,21,22,23,25,35,41,42,43,48]. Detailed quality assessment results are provided in Table S2.

### 3.3. Prevalence of Sarcopenic Obesity

**Figure 2:**
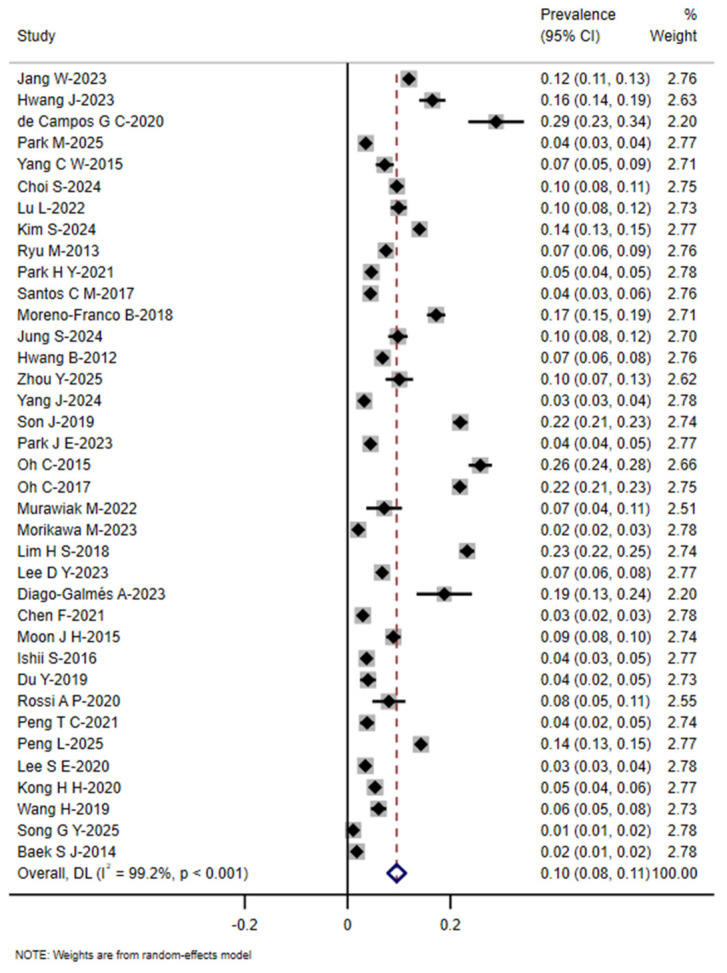
Forest plot of the prevalence of sarcopenic obesity in community-dwelling older adults [6,7,8,9,10,11,21,22,23,24,25,26,27,28,29,30,31,32,33,34,35,36,37,38,39,40,41,42,43,44,45,46,47,48,49,50,51].

Among the 37 studies available for meta-analysis, the prevalence of SO in community-dwelling older adults ranged from 1.08% to 29.3%. Using a random-effects model, the pooled prevalence was 10% (95%*CI*: 8–11%). The corresponding forest plot is presented in Figure 2. Due to the substantial heterogeneity across studies (*I*^2^ = 99.2%, *p* < 0.001), the 95% prediction interval for the prevalence of SO in community-dwelling older adults was estimated to be 1.4% to 31.2%.

### 3.4. Subgroup and Meta-Regression Analyses of Prevalence

Subgroup analyses were conducted based on country development level, region, study design, gender, assessment method for muscle mass, and diagnostic criteria for sarcopenia and obesity. The results showed that substantial heterogeneity remained within each subgroup, as presented in Table 1. To further explore potential sources of the observed variability in prevalence estimates across studies, univariate meta-regression analyses were performed. The results indicated that the adjusted *R*-squared (Adj *R*^2^) values for all included covariates were below 5%, and the residual *I*-squared (*I*^2^_res) values exceeded 99%, suggesting that these factors did not substantially account for the high between-study heterogeneity. Moreover, none of the covariates had a significant effect on the prevalence of SO among community-dwelling older adults (all *p* > 0.05). Detailed results are shown in Table S3.

### 3.5. Influencing Factors

A meta-analysis was conducted on 13 potential associated factors. The results indicated that the associations between SO and age, gender, physical activity, income level, unemployment, and energy intake level reached statistical significance. Due to insufficient data, some factors could not be included in the meta-analysis and were therefore described qualitatively. For example, one study identified that community-dwelling older women had a higher risk of SO [35]. Two studies suggested that sleeping more than 9 h per day may increase the risk [7,27]. Another study found that coffee consumption was associated with a lower prevalence of SO [40]. In addition, dietary patterns were also observed to be relevant, with one study indicating that a lacto-ovo-vegetarian dietary pattern might reduce the risk of SO among community-dwelling older adults [42]. The analysis of associated factors is detailed in Table 2.

### 3.6. Prognosis

Given that the number of available studies reporting on the prognosis of SO in community-dwelling older adults was limited, a quantitative meta-analysis was not feasible; we only performed a descriptive summary. The findings indicated that adverse outcomes associated with SO included falls [46], disability [21], osteoporosis [21,45], malnutrition [38], decreased pulmonary function [43,48], cognitive impairment [9,47,50], decline in physical function [8,49] and quality of life [8,9], negative emotional states such as depression and anxiety [9,21,44], increased risk of hospitalization [22], and even mortality [35]. Additionally, it was noteworthy that SO was also correlated with the development of comorbidities, such as heart disease [9,21], hypertension [9], diabetes [39], stroke [9], and hyperlipidemia [39,43,49].

### 3.7. Sensitivity Analysis

**Figure 3:**
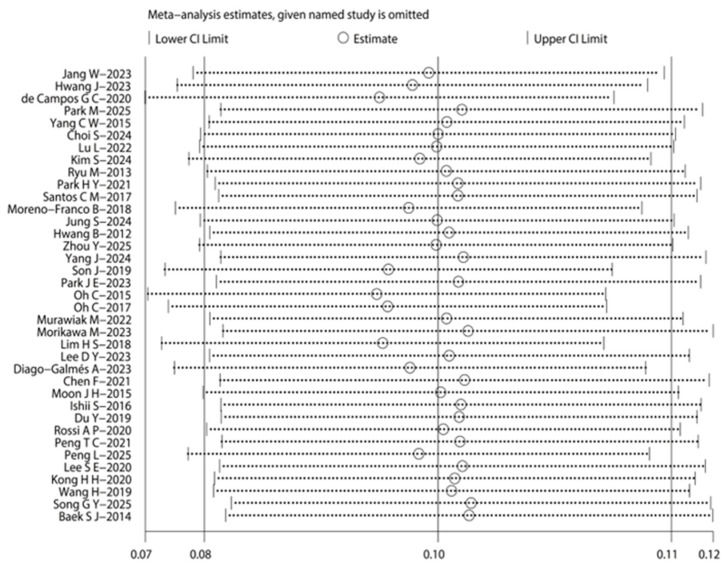
Sensitivity analysis of reported prevalence studies [6,7,8,9,10,11,21,22,23,24,25,26,27,28,29,30,31,32,33,34,35,36,37,38,39,40,41,42,43,44,45,46,47,48,49,50,51].

Sensitivity analysis based on model switching revealed that apart from insufficient protein intake and the presence of chronic diseases, most other associated factors or the prevalence estimates showed no significant changes, as detailed in Table 3. Additionally, a leave-one-out sensitivity analysis was performed on studies reporting prevalence. The results demonstrated that no single study exerted an undue influence on the overall estimates, verifying the stability of the results, as illustrated in Figure 3.

### 3.8. Publication Bias

**Figure 4:**
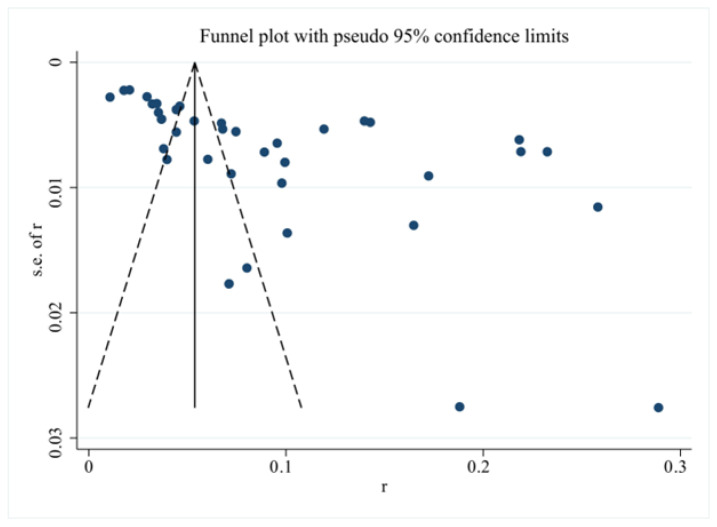
Funnel plot for assessing publication biases.

Given that the number of studies included for each influencing factor was below 10, only publication bias was tested for prevalence. Asymmetry in the funnel plot revealed publication bias in the included studies (see Figure 4), which was further confirmed by Egger’s test (*p* < 0.10).

## 4. Discussion

This study analyzed the prevalence, associated factors, and prognosis of SO in community-dwelling older adults. Through a comprehensive search of domestic and international databases, a total of 37 studies involving 80,337 community-dwelling older adults aged ≥60 years were finally included. The results indicated that a pooled prevalence of SO in this population was 10%, with a 95% prediction interval ranging from 1.4% to 31.2%, indicating substantial heterogeneity in the true prevalence across studies. Analysis of associated factors revealed that SO was associated with six factors, including physical activity intensity, age, gender and so on. However, due to the limited number of prognostic studies, only descriptive methods were used for prognostic analysis. The findings suggested that SO adversely affects both physical and psychological health in older adults and may increase mortality risk.

This meta-analysis synthesized data on the prevalence of SO among older adults in the communities worldwide and identified a high degree of heterogeneity across the included studies. To analyze the source of heterogeneity, subgroup analyses and meta-regression were performed based on factors such as muscle mass assessment tools, diagnostic criteria for SO, and geographic region. Despite these efforts, considerable heterogeneity remained, suggesting that the variability may stem from differences in diagnostic criteria, study design, and population characteristics across studies. Further research is warranted to identify the specific sources of heterogeneity. The results of the subgroup analysis showed that the prevalence estimates of SO in the community-dwelling older adults differed between studies using bioelectrical impedance analysis (BIA) and those using dual-energy X-ray absorptiometry (DXA), which may be related to the inability of BIA to accurately determine muscle mass and fat mass. Current evidence indicates that DXA demonstrates high accuracy and safety in body composition measurement. In contrast, although BIA is fast and non-invasive, it tends to overestimate muscle mass [52] and underestimate fat mass [53]. In addition, BIA has different types of devices and models. Studies suggest that the results obtained by BIA using multi-frequency devices are closest to those of DXA [1]. The 2019 AWGS also recommends the use of multi-frequency BIA devices [17]. Therefore, in future studies, if conditions permit, the use of DXA may be considered for measurement. If BIA is used to evaluate muscle mass, a multi-frequency device may be used to improve the accuracy of the results. In terms of the diagnostic criteria for SO, the reported prevalence varies greatly due to the different diagnostic criteria adopted by each study. This mainly stems from the differences in the applicability of different standards to different groups of people and the accuracy of diagnosis. Our study found that when the obesity diagnosis criteria met any indicator such as WC, BF, BMI or TS, the prevalence of SO among community-dwelling older adults differed from that based on a single obesity indicator. This difference may arise from the inherent limitations of using a single obesity indicator. Previous evidence shows that for people with abdominal obesity but normal BMI, if obesity is diagnosed only based on BMI, the risk of visceral fat accumulation cannot be identified, which leads to missed diagnosis of the population. Such underdiagnosis directly reduces the number of identified SO cases, thereby leading to an underestimation of its prevalence [54]. Therefore, clinicians should select appropriate diagnostic criteria based on the age, gender, physical characteristics, and clinical profile of the target population. Where feasible, combining multiple diagnostic indicators for obesity is recommended to enhance diagnostic accuracy.

Additionally, this study found that the prevalence of SO among community-dwelling older adults varied across different study types, which may be related to the study design. For instance, one cohort study of 4197 participants reported a prevalence of only 2.07% [21]. This low figure may be attributable to the use of non-random sampling methods. Our study also revealed that the prevalence of SO among community-dwelling older adults differed between developed and developing countries as well as across different geographic regions. This may be attributed to inconsistencies in the diagnostic criteria and cut-off values for SO adopted across different countries, in addition to variations in sociodemographic characteristics between populations.

This study demonstrated that advanced age, male sex, physical activity level, low income, insufficient energy intake, and unemployment were associated with the occurrence of SO in community-dwelling older adults. The elderly aged 75 and above in the community had a high risk of SO. After the age of 50, muscle mass decreases annually due to a reduction in the number and size of muscle fibers. This change is primarily associated with the gradual loss of motor neurons [55]. Concurrently, fat mass tends to increase with age, peaking at the age of 65 to 75 [56]. Judging from the distribution of body fat, the fat storage gradually shifts from the periphery to the abdomen, resulting in a decrease in subcutaneous adipose tissue. This can lead to triglyceride overflow and lead to abnormal accumulation of muscle tissue, which eventually results in muscle dysfunction [57,58]. In addition, adipose tissue dysfunction associated with aging releases free fatty acids, which accumulate inside and outside the muscle fibers. Through oxidative stress, it produces lipotoxicity and aggravates muscle damage, thus promoting the occurrence of SO [59,60]. Therefore, primary medical institutions should strengthen the screening of SO in the elderly aged 75 and above in the community. During the screening process, DXA can be used, with a focus on the changing trend of muscle mass and visceral fat area.

The meta-analysis indicated that the elderly in communities with low physical activity levels were at an increased risk of SO, while moderate and high levels of physical activity can reduce the risk of SO. Evidence shows that reduced physical activity can lead to adipose tissue hypertrophy and chronic inflammation, and this chronic inflammatory state can exacerbate muscle tissue atrophy [61,62]. Numerous studies have indicated that regular resistance exercise can effectively reverse this process. It activates beneficial signaling pathways in muscle, and enhances metabolic function and regenerative capacity, thereby mitigating the negative impacts of fat accumulation, significantly improving muscle quality and function, and ultimately delaying the onset and progression of SO [63,64]. Research by Zhu et al. further confirms that the muscle strength and physical function of subjects practicing traditional health exercises (e.g., Tai Chi) for eight weeks were significantly improved [65]. Therefore, it is recommended that community-dwelling older adults combine resistance training with traditional health exercises within a structured exercise plan. Primary healthcare institutions can promote such plans by organizing community health lectures and regular group exercise sessions. All exercise should be conducted under the guidance of professionals and follow the principle of gradualism to avoid injury. Simultaneously, community-dwelling older adult males had a higher risk of SO. In males, serum testosterone levels decrease by approximately 2–3% per year with age [66]. This downward trend may lead to reduced muscle mass [67] and fat accumulation [68], thus increasing the risk of SO. Due to insufficient data, we did not conduct a meta-analysis specific to the female group. Therefore, future research still needs to conduct in-depth and systematic research focusing on female populations.

Furthermore, low income and unemployment are associated with an increased risk of SO among community-dwelling older adults. Income level is a key factor affecting nutritional status. Nutritional imbalance and malnutrition are closely linked to obesity and decreased muscle mass in the elderly [23]. Notably, studies have found that the relationship between obesity and socioeconomic status varies across countries with different income levels. In low- and middle-income countries, obesity is more common among individuals with a higher socioeconomic status, whereas in high-income countries, it is more prevalent among those with a lower socioeconomic status [23]. Therefore, it is recommended that policymakers develop targeted social policies. These policies should integrate economic assistance with health promotion, including providing certain nutritional subsidies to low-income groups and systematically disseminating the knowledge on the prevention and treatment of SO.

We found that insufficient energy intake was associated with the occurrence of SO among community-dwelling older adults. Energy supply is a key factor in maintaining muscle function. Although a low-calorie diet may aid in weight loss, chronic insufficient energy intake adversely affects muscle mass and function [69]. Sustained insufficient energy intake can prompt the body to break down muscle protein for energy, and when the rate of protein degradation exceeds the rate of protein synthesis, muscle mass gradually declines, thereby exacerbating muscle atrophy [70]. Furthermore, multiple studies have shown that older adults with a daily protein intake of 1.1 g/kg body weight have a lower rate of muscle loss compared to those with a daily intake of 0.7 to 0.9 g/kg body weight [71,72,73]. Therefore, it is recommended that community-dwelling older adults adopt a balanced diet in their daily eating habits to ensure adequate energy and protein intake. For community-dwelling older adults aged 65 years and above, the optimal daily protein intake can be increased to 1.0–1.2 g/kg body weight to promote muscle regeneration, prevent muscle breakdown, and thereby maintain muscle health [74].

This study also found that SO can adversely affect physical function, metabolic health, and psychological status in community-dwelling older adults. In the study by Tao-Chun et al., the risk of falling in patients with SO increased significantly (OR = 3.33) [46]. Research by Yanping et al. reported that the risk of hyperglycemia in the SO group (OR = 5.65) was significantly higher than that in the simple obesity group (OR = 3.99) [45], suggesting that SO may further aggravate the occurrence of hyperglycemia. At the same time, we also observed that the prevalence of depression in this patient group was as high as 26.6%, significantly exceeding that in the non-sarcopenic obesity group [44]. However, at present, primary medical institutions have not paid sufficient attention to SO. Therefore, it is recommended that healthcare institutions establish a comprehensive intervention system from community screening to multidisciplinary management. Simple screening methods including calf circumference measurement, grip strength testing, and the SARC-F questionnaire should be integrated into primary care. Suspected cases should be referred to specialized units for precise assessment of muscle mass. Additionally, a multidisciplinary team composed of clinicians, nutritionists, and rehabilitators should be established to formulate individualized management plans covering regular exercise, nutritional supplementation, and psychological monitoring. Community follow-up and mobile health technology are necessary for effectively preventing the occurrence of SO and improving its adverse outcomes.

## 5. Strengths and Limitations of the Study

Our meta-analysis and systematic review have several strengths. First, our study systematically analyzed the prevalence, associated factors, and prognosis of SO among community-dwelling older adults. In this study, we conducted a comprehensive search of nine English and Chinese databases, with two researchers independently screening studies based on predetermined eligibility criteria to enhance the comprehensiveness and accuracy of the findings. Furthermore, sensitivity, meta-regression and subgroup analyses were performed to explore possible causes of heterogeneity and make the results more rigorous. However, our study also has several limitations. First, since most of the included studies were cross-sectional studies in design, this may introduce some bias. Owing to the limited number of studies on the prognosis of SO in this population, only a descriptive synthesis was possible; quantitative meta-analysis could not be performed. Finally, due to publication and language constraints, all included studies were published in English. Future studies need to overcome these limitations to further improve the accuracy and reliability of the research.

## 6. Conclusions

This systematic review evaluated the prevalence of SO in the elderly in the community, and analyzed the associated factors and prognosis of the disease, which can provide a theoretical foundation for managing and treating SO. Factors such as low physical activity, low income, and advanced age were associated with the occurrence of SO in community-dwelling older adults. Based on the above findings, future prevention and control strategies should focus on populations with these risk profiles by implementing targeted early interventions to reduce the risk of adverse outcomes.

## Figures and Tables

**Table 1:** Heterogeneity analyses.

Subgroups	Number of Included Studies	Sarcopenic Obesity in the Elderly in the Community			
		Prevalence	95%*CI*	*I* ^2^	*p* Value
Country					
Developed countries	26	10%	8–12%	99.4%	<0.001
Developing countries	11	8%	5–11%	98.3%	<0.001
Region					
Asia	31	9%	7–11%	99.3%	<0.001
South America	2	17%	−7–40%	98.7%	0.177
Europe	4	13%	7–19%	93.2%	<0.001
Study design					
Cross-sectional study	35	10%	8–12%	99.2%	<0.001
Cohort study	2	5%	−1–11%	92.3%	0.104
Gender					
Male	31	8%	7–10%	97.0%	<0.001
Female	31	10%	8–12%	98.8%	<0.001
Assessment method for muscle mass					
DXA	19	12%	9–14%	99.3%	<0.001
BIA	13	6%	5–8%	97.5%	<0.001
Diagnostic criteria of sarcopenia					
EWGSOP	7	12%	7–16%	97.2%	<0.001
AWGS	12	6%	4–8%	98.6%	<0.001
Other	18	11%	8–14%	99.5%	<0.001
Diagnostic criteria of obesity					
WC	13	10%	7–13%	99.3%	<0.001
BF	11	9%	7–11%	97.4%	<0.001
BMI	8	9%	5–13%	99.5%	<0.001
WC/BMI	2	12%	8–16%	88.2%	<0.001
BF/VF	1	2%	2–3%	0.0%	<0.001
BF/BMI/WC/TS	1	19%	13–24%	0.0%	<0.001
BMI/WC/BF	1	4%	2–5%	0.0%	<0.001

Abbreviations: DXA: Dual-energy X-ray Absorptiometry; BIA: Bioelectrical Impedance Analysis; EWGSOP: European Working Group on Sarcopenia in Older People; AWGS: Asian Working Group for Sarcopenia; WC: Waist Circumference; BF: Body Fat Percentage; BMI: Body Mass Index; VF: Visceral Fat; TS: Triceps Skinfold Thickness.

**Table 2:** Associated factors of sarcopenic obesity in community-dwelling older adults.

No.	Risk Factors	Number of Included Studies	OR	95%*CI*	*I* ^2^	*p* Value
1	Age ≥ 75	4	1.83	1.21–2.76	91.9%	0.004
2	Male	3	3.38	1.53–7.49	79.5%	0.003
3	Low physical activity	4	1.56	1.13–2.16	56.8%	0.008
4	Moderate-to-high physical activity	4	0.62	0.51–0.77	55.8%	<0.001
5	Low income	3	1.71	1.04–2.83	68.8%	0.036
6	Residence (rural)	4	0.88	0.71–1.10	25.1%	0.278
7	Low level of education	3	1.18	0.79–1.76	69.8%	0.410
8	Smoking	4	1.01	0.62–1.66	51.0%	0.957
9	Drinking	4	0.93	0.62–1.38	0.0%	0.704
10	Insufficient protein intake	4	1.41	0.98–2.03	58.0%	0.064
11	Insufficient energy intake	3	1.23	1.02–1.50	0.0%	0.032
12	Chronic disease	3	1.47	0.57–3.81	80.7%	0.427
13	Unemployment	3	1.88	1.29–2.75	59.2%	0.001

**Table 3:** Sensitivity analysis results of prevalence and associated factors for sarcopenic obesity in community-dwelling older adults.

Prevalence/Risk Factors	Fixed Effects Model					Random Effects Model				Stability
	Combined Effect Estimate	95%*CI*	*Z* Value	*p* Value		Combined Effect Estimate	95 % *CI*	*Z* Value	*p* Value	
Prevalence	0.05	0.05–0.06	69.421	<0.001		0.10	0.08–0.11	10.767	<0.001	Stable
Age ≥ 75	1.24	1.18–1.31	8.214	<0.001		1.83	1.21–2.76	2.864	0.004	Stable
Male	2.71	1.98–3.69	6.277	<0.001		3.38	1.53–7.49	3.008	0.003	Stable
Low physical activity	1.64	1.36–1.97	5.171	<0.001		1.56	1.13–2.16	2.673	0.008	Stable
Moderate-to-high physical activity	0.70	0.63–0.79	−6.122	<0.001		0.62	0.51–0.77	−4.495	<0.001	Stable
Low income	2.22	1.78–2.78	7.007	<0.001		1.71	1.04–2.83	2.098	0.036	Stable
Residence (rural)	0.88	0.71–1.10	−1.085	0.278		0.89	0.68–1.17	−0.831	0.406	Stable
Low level of education	1.18	0.97–1.45	1.626	0.104		1.18	0.79–1.76	0.824	0.410	Stable
Smoking	1.07	0.77–1.48	0.384	0.701		1.01	0.62–1.66	0.054	0.957	Stable
Drinking	0.93	0.62–1.38	−0.380	0.704		0.93	0.62–1.38	−0.380	0.704	Stable
Insufficient protein intake	1.31	1.06–1.62	2.467	0.014		1.41	0.98–2.03	1.852	0.064	Unstable
Insufficient energy intake	1.23	1.02–1.50	2.144	0.032		1.23	1.02–1.50	2.144	0.032	Stable
Chronic disease	1.84	1.24–2.74	3.002	0.003		1.47	0.57–3.81	0.794	0.427	Unstable
Unemployment	1.72	1.37–2.18	4.596	<0.001		1.88	1.29–2.75	3.280	0.001	Stable

## Data Availability

The original contributions presented in this study are included in the article, and further inquiries can be directed to the corresponding author.

## Supplementary Materials

The following supporting information can be downloaded at https://www.mdpi.com/article/10.3390/nu18081267/s1, Table S1. The search strategy for each database; Table S2. The basic characteristics and quality evaluation results of the included literature; Table S3. Univariate meta-regression analysis.
