# Supplementary material for: Prevalence and Associated Factors of Sarcopenic Obesity in the Community Elderly: Meta-Analysis and Systematic Review
Source: Nutrients. 2026 Apr 16;18(8):1267. doi: 10.3390/nu18081267 (PMC13118367; doi:10.3390/nu18081267)
Supplement: Supplementary file 1 [file nutrients-18-01267-s001.zip › nutrients-4230639-supplementary.pdf]

**Table S1.** The search strategy for each database

| Number | Databases | Search terms |
|---|---|---|
| 1 | Web of Science | #1 TOPIC: sarcopenia OR TOPIC: sarcopenic<br>#2 TOPIC: Obesity OR TOPIC: sarcopenic obesity OR TOPIC: Obesity, Abdominal OR TOPIC: Body Weight<br>#3 TOPIC: Prevalence OR TOPIC: Incidence OR TOPIC: Epidemiology OR TOPIC: frequency OR TOPIC: influence factors OR TOPIC: associated factors OR TOPIC: influencing factor OR TOPIC: affecting factor OR TOPIC: risk factor OR TOPIC: predictor OR TOPIC: relevant factor OR TOPIC: related factor OR TOPIC: causality<br>#4#1AND#2AND#3 |
| 2 | Embase | #1('sarcopenia'/exp OR 'sarcopenia') AND[embase]/lim<br>#2 'sarcopenic':ti,ab,kw AND [embase]/lim<br>#3 #1 OR #2 |

| | | |
|---|---|---|
| | | #4 ('obesity'/exp OR 'obesity') AND [embase]/lim<br>#5 ('sarcopenic obesity':ti,ab,kw OR 'obesity, abdominal':ti,ab,kw OR 'body weight':ti,ab,kw) AND [embase]/lim<br>#6 #4 OR #5<br>#7 ('prevalence':ti,ab,kw OR 'incidence':ti,ab,kw OR 'epidemiology':ti,ab,kw OR'frequency':ti,ab,kw OR 'influence factors':ti,ab,kw OR 'associated factors':ti,ab,kw OR 'influencing factor':ti,ab,kw OR 'affecting factor':ti,ab,kw OR 'risk factor':ti,ab,kw OR 'predictor':ti,ab,kwOR'relevantfactor':ti,ab,kw OR 'related factor':ti,ab,kw OR 'causality':ti,ab,kw) AND [embase]/lim<br>#8 #3 AND #6 AND #7 |
| 3 | Pubmed | #1 "Sarcopenia"[Mesh]<br>#2 sarcopenic[Title/Abstract]<br>#3 #1OR #2 |

| | | |
|---|---|---|
| | | #4 "Obesity"[Mesh]<br>#5 ((sarcopenic obesity[Title/Abstract]) OR (Obesity, Abdominal[Title/Abstract])) OR (Body Weight[Title/Abstract])<br>#6 #4OR#5<br>#7 (((((((((((Prevalence[Title/Abstract]) OR (Incidence[Title/Abstract])) OR (Epidemiology[Title/Abstract])) OR (frequency[Title/Abstract])) OR (influence factors[Title/Abstract])) OR (associated factors[Title/Abstract])) OR (influencing factor[Title/Abstract])) OR (affecting factor[Title/Abstract])) OR (risk factor[Title/Abstract])) OR (predictor[Title/Abstract])) OR (relevant<br>#8 #3AND#6 AND #7 |
| 4 | Cochrane library | #1 MeSH descriptor: [Sarcopenia] explode all trees<br>#2 (sarcopenic):ti,ab,kw<br>#3 #1 or #2 |

| | | |
|---|---|---|
| | | #4 MeSH descriptor: [Obesity] explode all trees<br>#5 (sarcopenic obesity OR Obesity, Abdominal OR Body Weight):ti,ab,kw<br>#6 #4 or #5<br>#7 (Prevalence OR Incidence OR Epidemiology OR frequency OR influence factors OR associated factors OR influencing factor OR affecting factor OR risk factor or predictor OR relevant factor OR related factor or causality):ti,ab,kw<br>#8 #3 AND #6AND#7 |
| 5 | CINAHAL | #1 SU "Sarcopenia" OR TI (sarcopenia or sarcopenic) OR AB (sarcopenia or sarcopenic)<br>#2 SU "Obesity" OR TI (Obesity or sarcopenic obesity or Obesity, Abdominal or Body Weight) OR AB (Obesity or sarcopenic obesity or Obesity, Abdominal or Body Weight)<br>#3 TI (Prevalence or Incidence or Epidemiology or frequency or influence factors or associated factors or influencing factor or affecting factor or risk factor or predictor or relevant factor or related factor or causality) OR AB (Prevalence or Incidence or Epidemiology or frequency or |

| | | |
|---|---|---|
| | | influence factors or associated factors or influencing factor or affecting factor or risk factor or predictor or relevant factor or related factor or causality)<br>#4#1AND#2AND#3 |

**Table S2.** The basic characteristics and quality evaluation results of the included literature

| Auther (year) | Country | Study design | Age （years） | Sample size （M/F） | Assessment method for muscle mass | Definition of SO | Prevalence (%) SO（M/F） | Associated factors | Prognosis | Quality assessment |
|---|---|---|---|---|---|---|---|---|---|---|
| Jang W (2023)[23] | Korea | Cross-sectional study | ≥65 | 3690(1645/2045) | / | Grip strength and WC | 11.92%(6.63% / 16.19%) | 1,2,3,4,5,7,14 | / | 8 |
| Hwang J (2023)[24] | Korea | Cross-sectional study | 75-84 | 813(325/488) | DXA | EWGSOP and WC | 16.48%(16.31% / 16.60%) | / | / | 7 |
| de Campos G C (2020)[8] | Brazil | Cross-sectional study | ≥65 | 270(81/189) | DXA | EWGSOP and BF | 29.30%(33.33% / 27%) | 10,5,6,7 | / | 8 |
| Park M (2025)[7] | Korea | Cross-sectional study | ≥65 | 2118(1003/1115) | DXA | AWGS and WC | 3.54%(5.88% / 1.43%） | 2,3,4,5,6,10，11,12,13,14,15 | / | 6 |
| Yang C W (2015)[25] | China | Cross-sectional study | ≥65 | 844(448/396) | DXA | EWGSOP and BF | 7.23%(7.37% / 7.07%) | 1 | / | 8 |
| Choi S (2024)[26] | Korea | Cross-sectional study | 70-84 | 2071(1030/1041) | DXA | ESPEN and EASO guidelines | 9.56%(8.74% / 10.37%) | 8 | / | 7 |
| Lu L (2022)[27] | China | Cross-sectional study | ≥65 | 1407(581/826) | BIA | AWGS and BF | 9.95%(13.94% / 7.14%) | 1,11,15,16 | / | 7 |
| Kim S (2024)[28] | Korea | Cross-sectional study | ≥65 | 5458(2391/3067) | / | Grip strength and WC | 13.98%(8.62% /18.16%) | / | / | 7 |
| Ryu M (2013)[29] | Korea | Cross-sectional study | ≥65 | 2264(940/1324) | DXA | SMI and WC | 7.46%(7.45% / 7.48%) | 8 | / | 7 |

| Table S2. Cont | | | | | | | | | | |
|---|---|---|---|---|---|---|---|---|---|---|
| Auther (year) | Country | Study design | Age （years） | Sample size （M/F） | Assessment method for muscle mass | Definition of SO | Prevalence (%) SO （M/F） | Associated factors | Prognosis | Quality assessment |
| Park H Y (2021)[30] | Korea | Cross-sectional study | ≥65 | 3573(1533 /2040) | DXA | SMI and BF | 4.62%(4.11% / 5.00%) | 8 | / | 7 |
| Santos C M (2017)[31] | Brazil | Cross-sectional study | ≥65 | 1373(490/883) | / | Grip strength and BMI | 4.44%(3.27% / 5.08%) | / | / | 7 |
| Moreno-Franco B (2018)[32] | Spain | Cross-sectional study | ≥65 | 1730(808/922) | BIA | SMI and BF | 17.23%(18.19% / 16.38%) | 1,3 | / | 7 |
| Jung S (2024)[33] | Korea | Cross-sectional study | 70-84 | 950(459/491) | DXA | AWGS and WC | 9.79%(10.89% / 8.76%) | / | / | 7 |
| Hwang B (2012)[34] | Korea | Cross-sectional study | ≥60 | 2221(964/1257) | DXA | SMI and WC | 6.80%(6.12% / 7.32%) | 2,4,5,6,14 | / | 6 |
| Zhou Y (2025)[35] | China | Cross-sectional study | ≥60 | 487(204/283) | BIA | AWGS and WC or BMI | 10.06%(5.88%/13.07%) | 1,2,17 | 12 | 8 |
| Yang J (2024)[9] | China | Cross-sectional study | ≥60 | 2821(1398/1423) | BIA | AWGS and BF | 3.23%(3.58%/2.88%） | / | 5,7,8,11 | 7 |
| Son J (2019)[36] | Korea | Cross-sectional study | ≥65 | 3367 | DXA | SMI and WC | 21.92% | 7,8,12,13 | / | 5 |
| Park J E (2023)[10] | Korea | Cross-sectional study | ≥65 | 2971(1275/1696) | DXA | AWGS and WC | 4.44%(4.86% / 4.13%) | 7,12,13 | / | 7 |

| Table S2. Cont | | | | | | | | | | |
|---|---|---|---|---|---|---|---|---|---|---|
| Auther (year) | Country | Study design | Age（years） | Sample size（M/F） | Assessment method for muscle mass | Definition of SO | Prevalence (%) SO（M/F） | Associated factors | Prognosis | Quality assessment |
| Oh C (2015)[11] | Korea | Cross-sectional study | ≥60 | 1433(658/775 ) | DXA | SMI and BMI | 25.82%(19.60%/31.10%） | / | / | 7 |
| Oh C (2017)[37] | Korea | Cross-sectional study | ≥60 | 4452(1929/2523) | DXA | SMI and BMI | 21.83% | 7,12 | / | 7 |
| Murawiak M (2022)[38] | Poland | Cross-sectional study | ≥60 | 211(84/127) | BIA | EWGSOP and BF | 7.11%(5.95% / 7.87%) | / | 9 | 6 |
| Morikawa M (2023)[21] | Japan | Cohort study | ≥65 | 4197(1922/2275) | BIA | AWGS and BF or VF | 2.07%(1.61%/ 2.46) | / | 2,3,8,11 | 7 |
| Lim H S (2018)[39] | Korea | Cross-sectional study | ≥65 | 3492(1850/1642) | DXA | SMI and WC | 23.25%(17.08% /30.21%） | / | 11 | 7 |
| Lee D Y (2023)[40] | Korea | Cross-sectional study | ≥65 | 2661 | DXA | AWGS and WC | 6.73% | Coffee | / | 7 |
| Diago-Galmés A (2023)[41] | Spain | Cross-sectional study | ≥65 | 202(38/164) | BIA | EWGSOP and BF or BMI or WC or TS | 18.81%(28.95% / 16.46%) | / | / | 8 |
| Chen F (2021)[42] | China | Cross-sectional study | ≥60 | 3795 | BIA | SMI and WC | 3.50% | Eating patterns | / | 8 |

| Table S2. Cont | | | | | | | | | | |
|---|---|---|---|---|---|---|---|---|---|---|
| Auther (year) | Country | Study design | Age （years） | Sample size （M/F） | Assessment method for muscle mass | Definition of SO | Prevalence (%) SO（M/F） | Associated factors | Prognosis | Quality assessment |
| Moon J H (2015)[43] | Korea | Cross-sectional study | ≥65 | 1583(635/948) | DXA | SMI and BMI | 8.91%(7.87% / 9.60%) | / | 4 | 8 |
| Ishii S (2016)[44] | Japan | Cross-sectional study | ≥65 | 1731(875/856) | BIA | SMI and BF | 3.70%(3.66% / 3.74%) | / | 8 | 7 |
| Du Y (2019)[45] | China | Cross-sectional study | ≥65 | 631(213/418) | BIA | AWGS and BMI | 3.96%(6.98% /2.39%) | 11 | 3,11 | 8 |
| Rossi A P (2020)[22] | Italy | Cohort study | 68-78 | 274(97/177) | DXA | SMI and BF | 8.03%(8.25% / 7.91%) | / | 10 | 7 |
| Peng T C (2021)[46] | China | Cross-sectional study | ≥65 | 765 | BIA | EWGSOP and BMI or WC or BF | 3.79% | / | 1 | 6 |
| Peng L (2025)[47] | China | Cross-sectional study | ≥60 | 5320(2973/2347) | / | AWGS and BMI or WC | 14.26%(9.15% / 20.75%) | / | 5 | 7 |
| Lee S E (2020)[48] | Korea | Cross-sectional study | ≥60 | 3044 | / | EWGSOP and BMI | 3.45% | / | 4 | 8 |
| Kong H H (2020)[49] | Korea | Cross-sectional study | 70-84 | 2303(1091/1212) | DXA | SMI and WC | 5.34%(3.85% / 6.68%) | / | 6 | 7 |

| Table S2. Cont | | | | | | | | | | |
|---|---|---|---|---|---|---|---|---|---|---|
| Auther (year) | Country | Study design | Age (years) | Sample size (M/F) | Assessment method for muscle mass | Definition of SO | Prevalence (%) SO (M/F) | Associated factors | Prognosis | Quality assessment |
| Wang H (2019)[50] | China | Cross-sectional study | 60-92 | 945(465/480) | BIA | AWGS and BF | 6.03%(7.31% / 4.79%) | / | 5 | 7 |
| Song G Y (2025)[6] | Korea | Cross-sectional study | ≥65 | 1387(635/752) | BIA | AWGS and BMI | 1.08%(0.79% / 1.33%) | / | 6，7 | 8 |
| Baek S J (2014)[51] | Korea | Cross-sectional study | ≥65 | 3483(1466/2017) | DXA | SMI and BMI | 1.78%(2.18% / 1.49%) | / | 11 | 5 |

Abbreviations: SO: Sarcopenic Obesity; M: Male; F: Female; BIA: Bioelectrical Impedance Analysis; DXA：Dual-energy X-ray Absorptiometry; AWGS: Asian Working Group for Sarcopenia; EWGSOP: European Working Group on Sarcopenia in Older People; ESPEN: European Society for Clinical Nutrition and Metabolism; EASO: European Association for the Study of Obesity; SMI: Skeletal Muscle Index; WC: Waist Circumference; BF: Body Fat Percentage; TS: Triceps Skinfold Thickness; VF: Visceral Fat; BMI: Body Mass Index.

Influencing factors:1. Age ≥ 75 years; 2. Rural residence; 3. Low education level; 4. Low income; 5. Smoking; 6. Alcohol consumption; 7. Low physical activity; 8. Moderate to high physical activity; 9. Chronic diseases; 10. Male; 11. Inadequate protein intake; 12. Inadequate energy intake; 13. Unemployed; 14. Sleep duration > 9 hours; 15.BMI; 16. Female.

Prognosis:1. Falls; 2. Disability; 3. Osteoporosis; 4. Decline in pulmonary function; 5. Cognitive impairment; 6. Decline in physical function; 7. Decline in quality of life; 8. Negative mood; 9. Malnutrition; 10. High risk of hospitalization; 11. Complications; 12. Mortality.

**Table S3.** Univariate meta-regression analysis

| Covariates | Coefficient | 95%CI | *P* value | $I^2$_res | Adj $R^2$ |
|---|---|---|---|---|---|
| Country | | | | | |
| Developing countries(ref) | 0.00 | | | | |
| Developed countries | 0.15 | -0.50-0.80 | 0.639 | 99.99% | -2.20% |
| Region | | | | | |
| Asia(ref) | 0.00 | | | | |
| Europe | 0.55 | -0.74-1.85 | 0.392 | 99.99% | -0.70% |
| South America | 0.56 | -0.38-1.50 | 0.233 | 99.99% | 1.31% |
| Gender | | | | | |
| Female(ref) | 0.00 | | | | |
| Male | -0.07 | -0.54-0.40 | 0.773 | 99.98% | -1.55% |
| Study design | | | | | |
| Cohort study(ref) | 0.00 | | | | |
| Cross-sectional study | 0.68 | -0.62-1.97 | 0.296 | 99.99% | 0.36% |
| Assessment method for muscle mass | | | | | |
| BIA(ref) | 0.00 | | | | |
| DXA | 0.49 | -0.08-1.06 | 0.090 | 99.99% | 5.35% |
| Diagnostic criteria of sarcopenia | | | | | |
| AWGS(ref) | 0.00 | | | | |
| EWGSOP | 0.33 | -0.42-1.08 | 0.373 | 99.99% | -0.52% |
| Other | 0.34 | -0.25-0.92 | 0.250 | 99.99% | 1.01% |
| Diagnostic criteria of obesity | | | | | |
| BMI(ref) | 0.00 | | | | |
| WC | 0.24 | -0.38-0.85 | 0.443 | 99.99% | -1.12% |
| BF | 0.09 | -0.56-0.74 | 0.783 | 99.99% | -2.64% |
| WC/BMI | 0.55 | -0.75-1.85 | 0.399 | 99.99% | -0.76% |
